# Evaluation of burnout levels among healthcare staff in anesthesiology departments in Greece - Is there a connection with anxiety and depression?

**DOI:** 10.3934/publichealth.2024027

**Published:** 2024-04-19

**Authors:** Aikaterini Toska, Sofia Ralli, Evangelos C. Fradelos, Ioanna Dimitriadou, Anastasios Christakis, Viktor Vus, Maria Saridi

**Affiliations:** 1 Laboratory of Clinical Nursing, Department of Nursing, University of Thessaly, Larissa, Greece - Open Hellenic University, Patra, Greece; 2 School of Social Sciences, Open Hellenic University, Patra, Greece; 3 Laboratory of Clinical Nursing, Department of Nursing, University of Thessaly, Larissa, Greece; 4 Institute for Social and Political Psychology NAES Ukraine

**Keywords:** burnout, anxiety, depression, anesthesiology departments, health professionals

## Abstract

**Introduction:**

Healthcare workers in anesthesiology departments often experience burnout syndrome, which may be combined with anxiety and depression.

**Aim:**

The study aimed to assess the levels of burnout among nurses and physicians working in anesthesiology departments in public hospitals in Attica and to investigate a possible correlation between burnout, anxiety, and depression.

**Methodology:**

A cross-sectional study was conducted on physicians and nurses working in anesthesiology departments in public hospitals in Attica, Greece. A questionnaire was distributed electronically using the snowball sampling method, including questions about demographic characteristics, burnout, anxiety, and depression.

**Results:**

Physicians and nurses in anesthesiology departments were found to have moderate levels of burnout, and normal/low levels of anxiety and depression. More specifically, it was found that 2% of physicians and 14.4% of nurses had extremely elevated levels of burnout. On the other hand, 6.1% of physicians and 23.7% of nurses had high anxiety, while 6.1% of physicians and 15.5% of nurses had elevated levels of depression. Females (*p* = 0.008), staff aged 45–55 (*p* = 0.021), lower educational level (*p* = 0.025), nurses (*p* = 0.001), more than 21 years of service (*p* = 0.001), and having children (*p* = 0.008) were determinants of greater levels of personal burnout. Work-related burnout correlated with having children (*p* = 0.017), whereas client-related burnout was significantly higher for nurses (*p* = 0.002). In addition, a correlation was found between anxiety, depression, and increased levels of burnout (*p* = 0.000).

**Conclusions:**

As physicians and nurses working in anesthesiology departments have stressful jobs and work long hours, it is important to further study their physical, emotional, and mental exhaustion as well as psychological resilience levels.

## 1. Introduction

Health professionals report increased levels of depressive disorders in addition to high work stress and burnout [1]. The tendency to develop mental disorders is due to them being responsible for human lives and the responsibility to avoid acts or omissions that endanger patients. Some important determinants of mental disorders in health professionals have been reported, such as career–life conflicts, role responsibility, decision-making, making mistakes, and the threat of lawsuits from patients and their families [2],[3]. A study reports that healthcare professionals show high rates of both suicidal ideation and suicide, with objective evidence of elevated levels of mental distress due to the inherently stressful nature of the profession [4].

Demanding working conditions, exhausting hours and lack of sleep, a disturbed relationship with colleagues, and professional uncertainty play a decisive role in the development of psychiatric symptoms that, in the long term, are likely to lead to depression. It has been shown that almost 30% of physicians show signs of clinical depression during their training, a percentage that decreases as their professional rehabilitation improves [4],[5]. According to previous studies, nearly one-third of healthcare workers (HCWs), regardless of their role, are likely to be suffering from depression, with much higher rates of depression being observed among nurses and physicians. This leads to increased absenteeism and productivity loss, which has a great impact on the delivery of effective care [6],[7].

In addition, working in a work-intensive environment, the huge responsibility of patient care, managing emergencies, and being confronted daily with pain leads to health professionals experiencing intense stress that, many times, they cannot externalize and share [8]. Research reports that stress in healthcare professionals is mainly related to high workload or lack of material resources and is inextricably linked to low job satisfaction, as well as to a mismatch between job expectations and the actual work environment [9],[10].

Anxiety in healthcare professionals appears to be related to poor physical and mental health and is a key cause of workers adopting behaviors harmful to the body such as alcohol abuse, smoking, and substance addiction [11], as well as exhibiting aggressive behavior both toward patients and colleagues, making mistakes, avoiding taking responsibility for lower quality services, making many mistakes, and losing interest. Unmanaged chronic stress in the workplace is related to burnout syndrome, a syndrome that results from the physical, emotional, and mental exhaustion of the individual. Exhausted health professionals cease to have positive feelings of sympathy or respect for patients, but also show decreased job satisfaction, resulting in inferior quality of health services [12]. Rates of burnout symptoms differ between medical specialties [1]; the prevalence of burnout in anesthesiology is among the highest of all medical specialties with rates ranging from 10% to 40%, and increased rates of burnout in anesthesiologists working in critical care settings [13]. Given the elevated levels of burnout in anesthesia department workers, this paper attempted to investigate the correlation between burnout and the manifestation of symptoms of anxiety and depression in the physicians and nurses of anesthesia departments of public hospitals in Attica, Greece.

## 2. Materials and methods

### 2.1. Sample

The total sample of the study consisted of 195 health professionals (physicians and nurses) working in anesthesiology departments in public hospitals in the region of Attica, Greece. As the target population was difficult to reach, the snowball sampling method was utilized, taking advantage of the fact that one of the authors worked in an anesthesiology department. Initially, a Google Forms link for the questionnaire was sent to 43 colleagues via e-mail, and then participants in the study were kindly asked to share the link with other individuals who met the criteria. The survey was conducted between December 2021 and January 2022, and it took approximately 7–8 minutes to complete. Before filling out the questionnaires, the health professionals were informed about the study and provided written consent. To be eligible to participate, individuals had to meet the following criteria:

- To be a physician or nurse
- To work in the anesthesiology department
- To work in a public hospital based in Attica

Any other combination was automatically an exclusion criterion.

### 2.2. Research tools

#### 2.2.1. Copenhagen burnout inventory (CBI)

The level of burnout was investigated using the CBI, developed by Kristensen et al. (2005) [14]. The CBI consists of 19 questions and evaluates burnout in three different subscales:

Personal exhaustion: Assesses the degree of physical and psychological exhaustion the person experiences (questions 1–6).

Work-related exhaustion: Assesses the degree of physical and psychological exhaustion the individual perceives about work (questions 7–13).

Client-related exhaustion: Assesses the degree of physical and psychological exhaustion that is considered by the individual to be related to interaction with patients (questions 14–19).

The Greek version of the CBI was used in the present study. In reliability analysis, Cronbach's alpha indicates a high level of internal consistency (personal α = 0.878; work-related α = 0.790; and client-related α = 0.835).

#### 2.2.2. Hospital anxiety and depression scale (HADS)

The level of anxiety and depression was assessed using the psychometric hospital anxiety and depression scale (HADS) developed by Zigmond & Snaith, (1983) [15]. The HADS is a self-report assessment tool comprising 14 items on a scale of 0–3. It is created to measure anxiety and depression, with 7 items allocated for each subscale. The score of each subscale is obtained by adding up the respective 7 items, with a possible score range of 0–21.

The Greek version of the HADS was used. In the current study, Cronbach alpha reliability coefficients of the HADS subscales were high (anxiety α = 0.853; depression α = 0.836).

#### 2.2.3. Demographic and occupational characteristics of the sample

Additionally, eight items were used for recording socio-demographic and occupational characteristics (gender, age group, educational level, marital status, number of children, specialty, years of service, and working hours).

### 2.3. Statistical analysis

Data were analyzed using SPSS version 25. For socio-demographic and occupational items, frequencies and percentage distribution are presented. Regarding CBI subscales, individuals are categorized into different levels of burnout following manufacturers' instructions: no burnout or low level of burnout, moderate level of burnout, and high or severe level of burnout. Regarding HADS, according to the instructions, total scores for anxiety and depression place individuals as normal, borderline abnormal, or abnormal cases. Mean values were calculated for both CBI and HADS, whereas comparisons were based on the percentage distributions among levels or cases and socio-demographic and occupational characteristics. All variables were transformed in nominal or categorical form; thus, tests were performed using the Chi-square test (*χ^2^* test). The level of statistical significance was set at 0.05.

### 2.4. Morality and ethics

The postgraduate study program committee “Health Care Management” of Hellenic Open University approved the study under decision number issue 5/03–11–2022. The individuals who were part of the final sample had to choose if they accepted to participate in the study after receiving the relevant information. Only after that, they were able to proceed to the questionnaire form.

## 3. Results

### 3.1. Socio-demographic and occupational characteristics

As shown in Table 1, most of the respondents were female (almost 3 out of 4 professionals), aged 34–55 years old (almost 3 out of 4), with at least a university background (more than 3 out of 5), married or cohabiting (more than 7 out of 10), and with children (almost 7 out of 10). The sample was practically divided between physicians and nurses (98 and 97 individuals, respectively), with more than half having worked for at least 21 years in the hospital, and finally, more than 4 out of 10 working for at least 41 hours weekly. Table 1 also presents mean values for all burnout subscales.

**Table 1.** Socio-demographic and occupational characteristics.

**Item**	***N* (%)**	**Personal burnout (m)**	**Work-related burnout (m)**	**Client-related burnout (m)**
Sex				
· Male	49 (25.1%)	47.9	50.9	39.4
· Female	146 (74.9%)	55.7	54.4	37.2
Age				
· 23–33	23 (11.8%)	43.5	44.9	32.2
· 34–44	51 (26.1%)	52.6	52.1	37.6
· 45–55	93 (47.7%)	57.4	57.2	40.0
· 56–66	28 (14.4%)	52.1	50.6	35.4
Educational level				
· Up to secondary	27 (13.8%)	59.6	59.3	49.4
· Vocational	45 (23.1%)	58.9	56.3	41.1
· University	55 (28.2%)	49.8	49.7	33.2
· Master degree	48 (24.6%)	53.6	53.3	36.7
· PhD	20 (10.3%)	45.2	49.8	29.8
Specialty				
· Physician	98 (50.3%)	49.0	48.6	31.3
· Nurse	97 (49.7%)	58.5	58.4	44.3
Years of service				
· 1–10	44 (22.6%)	46.2	46.9	31.0
· 11–20	51 (26.1%)	51.7	51.7	36.8
· 21–30	67 (34.4%)	59.0	58.4	41.3
· 31–40	33 (16.9%)	56.3	55.0	41.3
Working hours (per week)				
· 0–40	111 (56.9%)	54.5	53.9	41.5
· 41 or more	84 (43.1%)	52.8	52.9	32.8
Marital status				
· Married or cohabiting	137 (70.6%)	55.2	54.6	36.3
· Single	38 (19.6%)	49.8	52.3	42.9
· Divorced	15 (7.7%)	48.3	46.9	36.9
· Widowed	4 (2.1%)	61.5	56.3	43.8
Children				
· Yes	133 (68.2%)	56.0	54.8	36.1
· No	62 (31.8%)	48.9	50.7	41.4

### 3.2. Burnout

The mean scores as well as the percentage distribution of the three subscales of burnout are presented in Table 2. It seems that for the present sample, moderate to severe levels reach 65.1% for personal burnout (m = 53.7 ± 17.1), 63.9% for work-related burnout (m = 53.5 ± 16.9), and 29.7% for patient-related burnout (m = 37.8 ± 20.6).

**Table 2.** Levels of burnout among the sample.

**Burnout levels percentage distribution**				
**Burnout subscale**	***Mean (SD)***	**No/low (score < 50)**	**Moderate (score 50–74)**	**High/severe (score 75–100)**
**Personal burnout**	53.7 (17.1)	34.9%	51.8%	13.3%
**Work-related burnout**	53.5 (16.9)	36.1%	50.5%	13.4%
**Client-related burnout**	37.8 (20.6)	70.3%	25.1%	4.6%

Table 3 displays the levels of burnout across all three categories and presents the correlation test results with the demographic and occupational characteristics of the sample. Regarding personal burnout, six items were found to be factors significantly associated: sex (higher levels of burnout for women), age (higher levels for older individuals), educational level (lower levels of burnout for higher educational levels), specialty (higher levels of personal burnout for nurses), years of service (higher levels of personal burnout for people having worked at least 21 years), and children (higher levels of personal burnout for individuals that have children).

As for work-related burnout, only the presence of children seems to be a determinant factor. More specifically, individuals with children present higher levels of work-related burnout compared with individuals without children. Finally, specialty and working hours are significantly associated with client-related burnout. Nurses sow higher levels of client-related burnout than physicians, while people working 40 or fewer hours per week record higher levels of client-related burnout.

**Table 3.** Levels of burnout among the sample (*n* = 195).

**Project**		**Personal burnout**				**Work-related burnout**				**Client-related burnout**			
		**No/low**	**Moderate**	**High/severe**	***Sig*.**	**No/low**	**Moderate**	**High/severe**	***Sig*.**	**No/low**	**Moderate**	**High/severe**	***Sig.***
**Sex**	Male	53.0%	38.8%	8.2%	**0.008**	42.8%	38.8%	18.4%	0.147	65.3%	26.5%	8.2%	0.356
	Female	28.8%	56.1%	15.1%		33.8%	54.5%	11.7%		71.9%	24.7%	3.4%	
**Age**	23–33	56.5%	43.5%	0.0%	**0.021**	56.6%	39.1%	4.3%	0.127	73.9%	26.1%	0.0%	0.871
	34–44	43.1%	47.1%	9.8%		44.0%	44.0%	12.0%		72.5%	21.6%	5.9%	
	45–55	23.7%	57.0%	19.3%		26.9%	55.9%	17.2%		66.7%	28.0%	5.3%	
	56–66	39.3%	50.0%	10.7%		35.7%	53.6%	10.7%		75.0%	21.4%	3.6%	
**Educational Level**	Up to secondary	22.2%	55.6%	22.2%	**0.025**	25.9%	55.6%	18.5%	0.906	63.0%	25.9%	11.1%	0.272
	Vocational	17.8%	68.9%	13.3%		35.6%	48.9%	15.5%		60.0%	33.3%	6.7%	
	University	45.5%	41.8%	12.7%		40.0%	49.1%	10.9%		80.0%	16.4%	3.6%	
	Master degree	37.5%	47.9%	14.6%		37.5%	47.9%	14.6%		68.7%	29.2%	2.1%	
	PhD	55.0%	45.0%	0.0%		36.8%	57.9%	5.3%		80.0%	20.0%	0.0%	
**Specialty**	Physician	45.9%	47.0%	7.1%	**0.001**	42.3%	49.5%	8.2%	0.051	80.6%	18.4%	1.0%	**0.002**
	Nurse	23.7%	56.7%	19.6%		29.9%	51.5%	18.6%		59.8%	32.0%	8.2%	
**Years of Service**	1–10	54.5%	43.2%	2.3%	**0.001**	52.3%	40.9%	6.8%	0.060	81.8%	15.9%	2.3%	0.422
	11–20	37.3%	54.9%	7.8%		38.0%	54.0%	8.0%		72.5%	23.6%	3.9%	
	21–30	19.4%	56.7%	23.9%		25.4%	56.7%	17.9%		65.7%	29.8%	4.5%	
	31–40	36.3%	48.5%	15.2%		33.3%	45.5%	21.2%		60.6%	30.3%	9.1%	
**Working hours**	0–40	33.3%	51.4%	15.3%	0.624	36.9%	48.7%	14.4%	0.807	63.1%	28.8%	8.1%	**0.006**
	41 or more	36.9%	52.4%	10.7%		34.9%	53.0%	12.1%		79.8%	20.2%	0.0%	
**Marital status**	Married or cohabiting	30.7%	56.2%	13.1%	0.377	30.9%	55.1%	14.0%	0.368	74.5%	22.6%	2.9%	0.070
	Single	47.4%	42.1%	10.5%		47.4%	39.5%	13.1%		60.5%	26.3%	13.2%	
	Divorced	46.7%	33.3%	20.0%		53.3%	40.0%	6.7%		60.0%	40.0%	0.0%	
	Widowed	25.0%	50.0%	25.0%		25.0%	50.0%	25.0%		50.0%	50.0%	0.0%	
**Children**	Yes	27.8%	56.4%	15.8%	**0.008**	29.6%	56.8%	13.6%	**0.017**	74.4%	22.6%	3.0%	0.106
	No	50.0%	41.9%	8.1%		50.0%	37.1%	12.9%		61.3%	30.6%	8.1%	

### 3.3. Anxiety and depression

The normal, borderline, and abnormal percentage distribution of depression and anxiety among the sample is shown in Table 4. There is a significant percentage of individuals that, according to HADS categorization, are considered borderline cases or cases for both anxiety (m = 6.9 ± 4.0) and depression (m = 7.1 ± 4.2), reaching 41.8% and 43.1%, respectively.

**Table 4.** Levels of anxiety and depression among the sample.

**Anxiety and depression percentage distribution**				
**Scale**	***Mean (SD)***	**Normal/*N* (%)**	**Borderline abnormal (borderline case)/*N* (%)**	**Abnormal (case)/*N* (%)**
**Anxiety**	6.9 (4.0)	113 (58.2%)	44 (22.7%)	37 (19.1%)
**Depression**	7.1 (4.2)	111 (57.2%)	42 (21.7%)	41 (21.1%)

Table 5 indicates that both anxiety and depression were found to be significantly associated with all three subscales of burnout, namely personal, work related, and client related.

**Table 5.** Spearman correlation between burnout categories, depression, and anxiety.

**No**	**Personal burnout (1)**	**Work-related burnout (2)**	**Client-related burnout (3)**	**Depression (4)**	**Anxiety (5)**
1	1.000	0.761**	0.429**	0.569**	0.525**
2	0.761**	1.000	0.580**	0.466**	0.415**
3	0.429**	0.580**	1.000	0.320**	0.270**
4	0.569**	0.466**	0.320**	1.000	0.667**
5	0.525**	0.415**	0.270**	0.667**	1.000

Note: **Correlation is significant at the 0.01 level (2-tailed).

## 4. Discussion

In this cross-sectional study, we delve into the dynamics of burnout, anxiety, and depression among healthcare professionals working in anesthesiology departments in Attica, Greece. Our findings reveal a noteworthy pattern, as both physicians and nurses exhibit moderate levels of burnout alongside comparatively lower levels of anxiety and depression. Of particular interest is the discernible correlation that emerges, highlighting the interconnected nature of anxiety, depression, and professional burnout within this healthcare cohort. Given the documented elevated levels of burnout among workers in anesthesiology departments, the primary objective of this paper is to systematically investigate the correlation between burnout and the manifestation of symptoms associated with anxiety and depression among physicians and nurses working in the anesthesiology departments of public hospitals in Attica.

The prevalence of burnout among healthcare workers in the anesthesiology department is a concern, as evidenced by numerous studies shedding light on the alarming rates of psychological distress within this professional cohort. Magnavita et al (2020) [16] uncovered a distressing reality, reporting that 51% of anesthesiologists experienced depression, with 27.8% grappling with anxiety. Notably, 71.1% of those facing anxiety also reported elevated stress levels, primarily linked to the demanding nature of their work and a perceived lack of adequate reward. Romito et al (2020) [9] delved into occupational burnout among physicians and nurses in anesthesia departments, revealing a substantial 40% prevalence. The identified risk factors included environmental social isolation, extensive working hours, and a perceived lack of control over the work environment. This resonates with the findings of Del Grosso and Boyd (2019) [17], who highlighted that nurse anesthetists often grapple with elevated levels of burnout attributed to intense workloads, fear of making errors, and a perceived lack of control.

Unveiling the intricacies of the mental health landscape within anesthesiology departments, our analysis delineates a delicate equilibrium in the psychological fortitude of both physicians and nurses. The moderate levels of burnout illuminate the pervasive challenges inherent to these roles, indicative of the toll exacted by the demanding, time-sensitive nature of their duties. Yet, the incongruent presence of relatively low levels of anxiety and depression beckons a deeper exploration into potential coping mechanisms or adaptive responses specific to this medical milieu. Remarkably, our study aligns with the findings reported by Briciu et al. in 2020 [18], revealing a similar prevalence of moderate and severe burnout. That study focused on 1052 healthcare professionals on the frontline of COVID-19 and documented burnout in 56.1% of participants. This synchronicity in burnout prevalence across different healthcare settings and specialties underscores the pervasive impact of the demanding nature of healthcare work, especially heightened during the challenging circumstances brought about by the COVID-19 pandemic.

In contrast to anticipated outcomes, our study revealed that gender, marital status, age, the presence of children, and weekly working hours exhibited no significant associations with burnout, anxiety, or depression among participants. However, this departure from expectations contrasts with established literature, where it is commonly asserted that the female gender is more susceptible to anxiety, depression, and burnout. Additionally, prevailing studies often suggest a direct impact of both age and the presence of children on mental health outcomes [19],[20]. The departure from these well-established patterns in our findings may be attributed to a notable gender imbalance, where women significantly outnumbered men. Adding to the complexity of our findings, Wang et al. (2020) [21] identified healthcare professionals over 29 years of age as experiencing the greatest burnout, while Zarei and al (2019) [22] reported that health workers over 35 years old have the highest burnout rates. These observations underscore the need for a more nuanced exploration of demographic factors in mental health research, as the dynamics within our participant cohort may have influenced the outcomes.

However, an interesting dichotomy emerges between physicians and nurses, with nurses manifesting higher levels of burnout, anxiety, and depression in comparison to their medical counterparts. This is not an unexpected finding, since literature shows that nurses are at increased risk of burnout in the light of the position they have within the working organization and the tasks they are generally assigned. This observed contrast aligns with findings from other studies, notably the research conducted by Izdebski et al [23]. Their study underscored the vulnerability of nurses, particularly in comparison with physicians. In the context of high-stress and demanding intensive care unit (ICU) settings, nurses emerged as a group disproportionately affected, reporting elevated levels of stress, burnout, and notably lower job satisfaction. The identified stressors for nurses in such environments were multifaceted. Personnel issues and work overload emerged as pervasive stressors, considering the acute challenges nurses face in their daily professional lives [24].

Previously published studies on healthcare workers' well-being consistently highlight diverse job risk factors, particularly noting that nurses, being under 40 years old, and those with longer working hours tend to experience poorer outcomes [10],[25]. Our data reveals associations between higher levels of burnout and factors such as educational level and years of experience. Our research aligns with this body of evidence, shedding light on the intricate associations between elevated burnout levels and distinct variables such as educational attainment and years of professional experience. Moreover, our findings resonate with the work of Lai and colleagues (2020) [26], as we observed a parallel correlation between higher educational levels and diminished levels of anxiety, depression, and burnout among healthcare workers. This concordance in outcomes strengthens the robustness of the evidence supporting the notion that a higher educational background serves as a protective factor against the deleterious effects of workplace stressors in the healthcare domain. Moreover, our study identifies a consistent link between burnout and years of experience, seamlessly aligning with existing literature. This reaffirms the idea that the cumulative effects of prolonged exposure to high-stress environments significantly contribute to heightened professional exhaustion among healthcare professionals [27].

Confirming intricate links between burnout, anxiety, and depression, our research underscores the call for holistic interventions. Consistent with global studies, we establish burnout because of anxiety and a significant risk factor for depression [28]–[30]. This recognition emphasizes the need for proactive measures to address stress and burnout, preventing their progression to more severe mental health conditions [31]. The bidirectional impact of depression and anxiety on burnout further accentuates the interconnected nature of these challenges. Advocating for integrated interventions, our findings stress the importance of a comprehensive approach for a more effective response to these complex mental health dynamics.

## 5. Limitations

Our study, while offering valuable insights, is subject to certain limitations that warrant consideration. Firstly, the study's focus on anesthesiology health professionals in Attica, Greece, may limit generalizability to broader healthcare contexts or diverse cultural settings. The noticeable gender imbalance within our participant cohort underscores the need for caution when extrapolating gender-related findings. The study also did not extensively explore specific work environment factors or coping mechanisms, suggesting avenues for future research. Furthermore, the impact of COVID-19 on mental health, while implicitly acknowledged, was not explicitly investigated. Despite these limitations, our study provides a foundation for understanding mental well-being in anesthesiology professionals, encouraging further research and targeted interventions.

## 6. Conclusion

The present study reveals that anesthesiology health professionals experience moderate levels of burnout. Factors such as gender, age, and number of children may be significant predictors of personal burnout. A difference is noted between physicians and nurses, with nurses experiencing higher levels of personal and client-related burnout, which is consistent with existing research on their vulnerability in high-stress environments. Educational level and experience also affect personal burnout, reflecting global research trends. A significant association among all subcategories of burnout with anxiety and depression was found. Thus, recognizing the interconnected nature of mental health challenges, the present study advocates for holistic interventions. Tailored strategies are crucial for this healthcare cohort, emphasizing the need for resilience and support in an ever-evolving healthcare landscape.

## Use of AI tools declaration

The authors declare they have not used Artificial Intelligence (AI) tools in the creation of this article.
